# The Multiple Functions of Melatonin: Applications in the Military Setting

**DOI:** 10.3390/biomedicines11010005

**Published:** 2022-12-21

**Authors:** Giuseppe Gancitano, Russel J. Reiter

**Affiliations:** 1 1st Carabinieri Paratrooper Regiment “Tuscania”, Italian Ministry of Defence, 57127 Livorno, Italy; 2 Department of Cell Systems and Anatomy, UT Health, Long School of Medicine, San Antonio, TX 78229, USA

**Keywords:** melatonin, military combat personnel, heart rate variability, HRV

## Abstract

The aim of this review is to provide the reader with a general overview on the rationale for the use of melatonin by military personnel. This is a technique that is being increasingly employed to manage growing psycho-physical loads. In this context, melatonin, a pleotropic and regulatory molecule, has a potential preventive and therapeutic role in maintaining the operational efficiency of military personnel. In battlefield conditions in particular, the time to treatment after an injury is often a major issue since the injured may not have immediate access to medical care. Any drug that would help to stabilize a wounded individual, especially if it can be immediately administered (e.g., per os) and has a very high safety profile over a large range of doses (as melatonin does) would be an important asset to reduce morbidity and mortality. Melatonin may also play a role in the oscillatory synchronization of the neuro–cardio–respiratory systems and, through its epigenetic action, poses the possibility of restoring the main oscillatory waves of the cardiovascular system, such as the Mayer wave and RSA (respiratory sinus arrhythmia), which, in physiological conditions, result in the oscillation of the heartbeat in synchrony with the breath. In the future, this could be a very promising field of investigation.

## 1. Introduction

Melatonin is an antioxidant/anti-inflammatory agent estimated to be 3 billion years old, and it probably evolved in bacteria. The blood levels of melatonin, which exhibit a circadian rhythm, are derived from the pineal gland. These levels are defined as central endogenous melatonin. One of the major actions of this kind of melatonin is influencing 24-h variations in organismal physiology. Additionally, however, there is also peripheral endogenous melatonin, which is produced in the mitochondria of many—perhaps all—cells. The main action of peripheral melatonin is likely to protect the mitochondria from damage due to locally generated oxygen and nitrogen-free radicals. Melatonin, together with its metabolites, which are also radical scavengers, is a powerful antioxidant present in all living organisms, including animals and plants. The potent antioxidant and anti-inflammatory actions of melatonin are the qualities that we feel make it highly useful to military personnel. Military operatives work in challenging and often complex scenarios, and must perform and recover as quickly as possible. Melatonin could have a potential role in performance and recovery processes. This review combines scientific evidence in support of the possible application of melatonin in combat and post-combat situations in order to improve individual resilience.

In particular, this review aims to examine the following possible applications of melatonin to support military personnel:

- As a protection from chemical and radioactive weapons;
- For the oscillatory synchronization of the neuro–cardio–respiratory systems;
- To support septic patients;
- To support critically traumatized patients.

## 2. Metal-Chelating Activity of Melatonin as Protection from Chemical and Radioactive Weapons

### 2.1. Chelating Activity of Melatonin: An Overview

Environmental pollution is considered a significant threat to human health due to the toxic effects of a variety of organic and inorganic pollutants [1]. Heavy metals comprise a major category of toxic environmental pollutants. An increasing body of data demonstrates that armed conflicts and military activity contribute significantly to environmental pollution [2]. A significant accumulation of metals has been observed in battlefields areas, small-arm shooting ranges, artilleries, mortar and rocket ranges, and grenade courts [3]. Military-related metal emissions therefore pose significant health hazards to military personnel, with the potential for overexposure. Occupational exposure to heavy metals can lead to the onset of various diseases, such as cardiovascular problems, neuronal damage, kidney damage, and an increased risk of cancer and diabetes.

### 2.2. Melatonin to Counteract Cellular Toxicity Induced by Heavy Metals

There are several mechanisms by which heavy metals induce cellular toxicity, but the main mechanism is the production of reactive oxygen species (ROS) that cause cellular oxidative damage [4]. Melatonin is highly effective in reducing such oxidative stress, as proven in a large number of reports. Its protective actions are the result of several means: the direct detoxification of ROS and reactive nitrogen species (RNS), the stimulation of antioxidant enzymes, and the suppression of other free-radical-generating enzymes. Additionally, melatonin also chelates transition metals, which are involved in the Fenton/Haber–Weiss reactions; due to its metal-chelating ability, melatonin reduces the formation of the highly reactive hydroxyl radical (•OH), which probably accounts for more than 50% of the oxidative stress that cells sustain [5]. Evidence shows that melatonin reacts with a number of metal ions, resulting in the formation of stable metal complexes. For example, using absorption voltammetry as a means of evaluation, it has been shown that melatonin, in proportion to its concentration, binds several heavy metals, including aluminum, cadmium, copper, iron, lead, and zinc, similar to metallothionein [6]. Melatonin chelates both iron (III) and iron (II), participating in the Fenton reaction to generate •OH. Melatonin is able to restore the biological activity of hemoglobin by acting on highly covalent iron and transforming it into iron (III), and it also reduces •OH, which is highly toxic.

### 2.3. Melatonin in Cerebrospinal Liquid (CSF): A Very Powerful Antioxidant

The important biochemical actions of melatonin are more effective than those carried out by metallothioneins, as the latter, being proteins, could be damaged by free radicals when they bind to transition metals. In comparison, melatonin neutralizes the generated free radicals, and thereby limits molecular and cellular damage. This is especially important in the brain, where metallothionein functions as a reducing agent when it binds metals. High levels of melatonin in the cerebrospinal fluid (CSF), and, consequently, in the brain, are important to ensure adequate protection against regional oxidative stress. In the brain, melatonin, via its direct scavenging activity and indirect antioxidant actions, significantly reduces the neural accumulation of oxidatively damaged molecules and seemingly compliments or replaces metallothionein as the major binder of transition metals.

### 2.4. The Journey of Melatonin in the Human Brain

Historically, the direct release of pineal melatonin into the rich pineal capillary bed has been accepted as the primary route of secretion. In reference to the central nervous system, however, the most important route of melatonin delivery may be its release via the pineal recess directly into the CSF of the third ventricle (3V). From the 3V, melatonin eventually moves with the CSF into the subarachnoid space that surrounds both the brain and spinal cord from where it has ready access to the neural parenchyma. Concentrations of melatonin in CSF increase rapidly at the onset of darkness and drop dramatically in the presence of light. Significant quantities of free radicals are produced in the brain due to the high metabolic demands of this organ, and in particular due to the high demand for oxygen, the parent molecule that produces numerous free radicals from its metabolism. The elevated levels of melatonin in the CSF, and therefore in the CNS, ensure shielding it from oxidative stress [7,8,9].

### 2.5. Melatonin as Copper’s Direct Chelator

**Figure 1:**
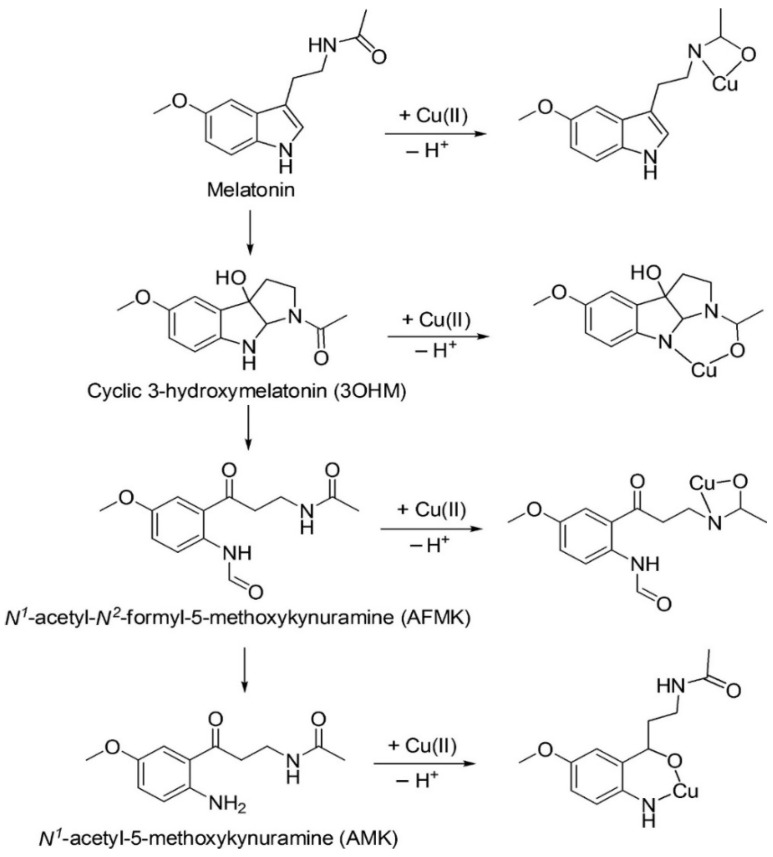
The free-radical-scavenging (vertical, **left**) and corresponding metal-chelating cascade (horizontal, **right**) of melatonin and its mentioned metabolites. The predicted chelation structures are those that are presumably the most abundant. The proposed mechanism, consistent with the predicted complexes, is a coupled deprotonation–chelation mechanism (CDCM). From Galano et al. [10], with permission.

The activity of melatonin and its metabolites cyclic 3-hydroxymelatonin (c3OHM), N1-acetyl-N2-formyl-5-methoxykinuramine (AFMK), and N1-acetyl-5-methoxykinuramine (AMK) in chelating metals has recently been analyzed [10]. When present in high intracellular concentrations, copper generates the •OH previously mentioned. Melatonin and its three metabolites produce stable complexes with copper ions (Figure 1). The direct chelation mechanism (DCM), compared with that of deprotonation–chelation, has been found to be a highly efficient chelation pathway of Cu (II) for melatonin and each of its metabolites. Furthermore, the melatonin/metabolite complex adequately interferes with the initial phases of the Haber–Weiss reaction, thus reducing the generation of highly oxidizing •OH. In consideration of these findings, Galano et al. [10] realized that melatonin, in addition to being the parent molecule in the free-radical-scavenging cascade [11], plays an essential role in metal chelation, as summarized in Figure 1.

### 2.6. Principal Mechanism of Metal Toxicity

A review was recently published describing the molecular damage to organisms caused by metals and the ability of melatonin to chelate these harmful substances [12]. This review summarized, in detail, the toxicity mechanisms of a number of heavy metals. The authors remind the reader that some metals are crucial to a wide variety of biological processes. In comparison, some xenobiotic metals, which lack physiological functions, interact with biological macromolecules and cause oxidative damage. These damaging agents include aluminum, cadmium, lead, mercury, and the metalloid arsenic. Although the molecular mechanisms are not fully understood, the main biochemical mechanisms by which heavy metals disrupt cellular homeostasis are described in significant detail [13].

#### 2.6.1. Cadmium and Melatonin

**Figure 2:**
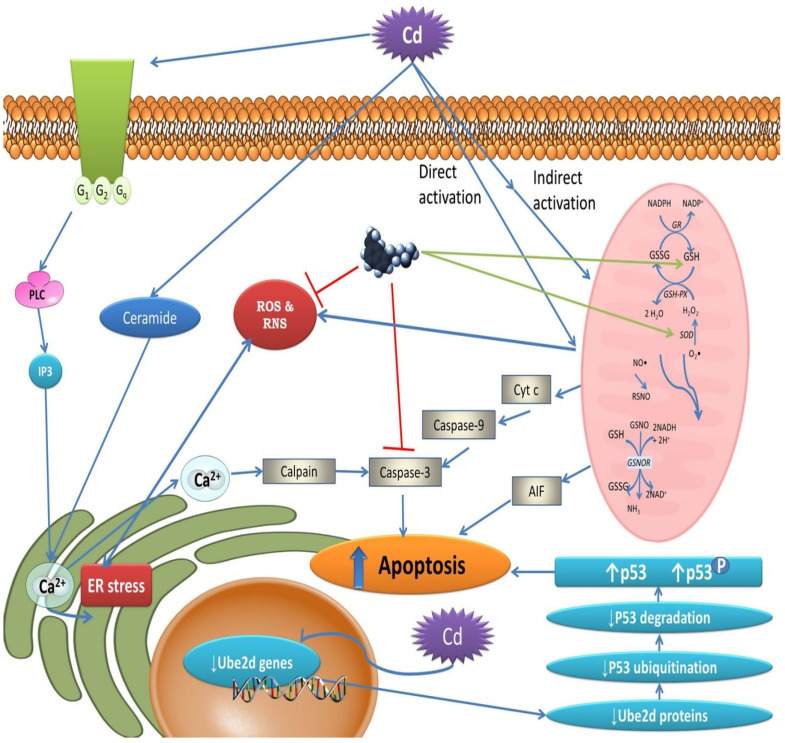
Presumptive cadmium pathways that cause cellular oxidative stress and leave damaged molecules in their wake. Cd induces endoplasmic reticulum (ER) stress by calcium overload through direct activation of ceramide and binding to protein G-coupled receptors. The increased intracellular Ca^2+^ induces calpain–caspase-3-mediated apoptosis. Cd both directly and indirectly promotes the formation of mitochondrial-derived ROS/RNS and releases the pro-apoptotic regulator cytochrome c (Cyt c), which stimulates caspase-3-mediated apoptosis. Cd downregulates Ube2d genes, thereby inhibiting P53 degradation, which causes P53 overload and the promotion of apoptosis. Melatonin counteracts Cd damage by blocking caspase-3 and reduces the ER stress caused by ROS/RNS. Melatonin also activates antioxidative enzymes and the GSH/GSSG cycle. From Romero et al. [12], with permission.

Cadmium (Cd^2+^) is a ubiquitous environmental contaminant that is ingested in drinking water and food and inhaled during breathing. High concentrations of cadmium are found in cigarettes, and Cd^2+^ is classified as a Class 1 human carcinogen [14]. At the cellular level, the main toxic effects induced by CD^2+^, as well as the potential sites of melatonin protection, are summarized in Figure 2.

In addition to direct scavenging and the indirect augmentation of the activities of antioxidative enzymes, in an in vivo study, melatonin was found to lower kidney Cd^2+^ accumulation [15]. This reduction in intracellular Cd^2+^ levels requires melatonin (i) to cross renal cell membranes [16], which it does due to its lipophilic character, permitting the removal of Cd^2+^ (ii) to establish stable complexes with Cd^2+^ or (iii) to the inhibit intestinal absorption of Cd^2+^, which would reduce the total body load of this damaging metal.

#### 2.6.2. Mercury and Melatonin

Mercury is a ubiquitous contaminant in the environment, making it very difficult for humans to avoid. It is considered a highly dangerous environmental pollutant. Humans and animals can come into contact with various forms of mercury, including elemental mercury (Hg) vapors, inorganic mercury [Hg (I)], mercury [Hg (II)], and organic mercury compounds [17]. There are several mechanisms by which these compounds alter the functioning of cells. Mercuric ions are able to bind to all molecules containing a thiol, such as glutathione (GSH), cysteine, metallothioneins (MTs), homocysteine, N-acetylcysteine (NAC), and albumin [18,19]. Consequently, the antioxidant cellular mechanisms resulting from exposure to this pollutant are altered [20].

#### 2.6.3. Melatonin against Mercury’s Neurotoxicity

Melatonin detoxifies numerous ROS including hydrogen peroxide (H_2_O_2_), hydroxyl radical (•OH), peroxyl radicals (ROO•), and singlet oxygen (^1^O_2_), in addition to RNS, including nitric oxide radicals (NO•) and peroxynitrite (ONOO−) [21]. Some or all of these are involved in the toxicity of mercury. The ability of melatonin to lower the neurotoxicity of mercuric chloride in animal studies has been shown. Similarly, since mercury poisoning is a proposed cause of Alzheimer’s disease [22], further documentation of the efficacy of melatonin in reducing mercury-induced neural damage has long-range implications for military personnel.

#### 2.6.4. Arsenic and Melatonin

**Figure 3:**
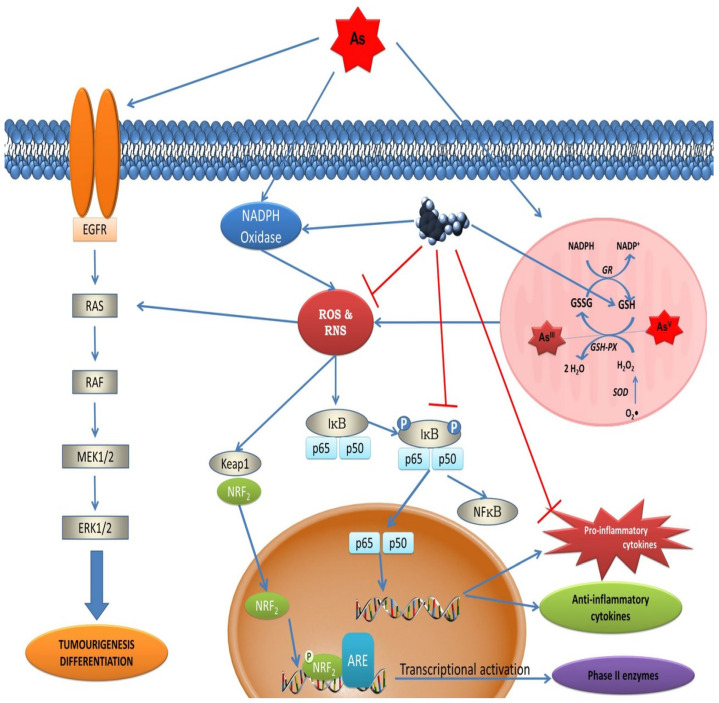
Arsenic-induced tumorigenesis and cell differentiation involve epidermal growth factor receptor (EGFR) signaling via MAP kinases. ROS generated after exposure to arsenic also activates molecules such as Ras and ERK and the IκB complex. IκB complex phosphorylation leads to p65 and p50 translocation into the nucleus, which causes the activation of pro- and anti-inflammatory cytokines. These are important contributors to carcinogenesis. ROS also activate nuclear factor (erythroid-derived 2)-like 2 (Nrf2), a key transcription factor that regulates the cellular redox response. The oxidation of As^3+^ to As^5+^ under physiological conditions leads to the formation of H_2_O_2_. Melatonin counteracts the toxic and tumorigenic effects caused by As by scavenging ROS production and stimulating antioxidative enzymes, increasing GSH levels, and modulating transcription factors such as NFκB and Nrf2. From Romero et al. [12], with permission.

The metalloid arsenic (As), which exists mainly in two biological oxidation states, arsenite [As (III)] and arsenate [As (V)], is a natural environmental contaminant with well-documented carcinogenic activity [23]; the trivalent forms of arsenic are considered the most dangerous [24]. They interact with sulfhydryl groups of proteins and inhibit many cellular biochemical reactions. The pentavalent forms are less toxic and act at the level of oxidative phosphorylation. Human beings can come into contact with both trivalent and pentavalent forms. The formation of numerous ROS/RNS in the metabolism of arsenic has been documented [25]. Consequently, the depletion of GSH due to consumption induced by the metabolism of As determines the establishment of an oxidizing intracellular environment, which is the basis for the process of carcinogenesis. Furthermore, reductions in antioxidant enzymes, including superoxide dismutase (SOD) and catalase (CAT), have been observed in vitro after exposure to As [26]. Melatonin reverses arsenic-induced metabolic toxicity by the means summarized in Figure 3.

#### 2.6.5. Lead and Melatonin

Lead is known to damage vital organs and suppress cellular processes. The major target organ of this heavy metal, in terms of toxicity, is the brain. The neurotoxicity of lead is expressed by altering the blood–brain barrier, astrocytes, and endothelia of the cerebral microcirculation [27]. Its accumulation occurs mainly at the hippocampal level, although all brain regions can be affected. Similarly to other heavy metals, pathogenetic actions are also multifactorial for lead, increasing the level of oxidative stress, altering the functioning of numerous enzymes, and inhibiting the absorption of trace elements, such as calcium and zinc [28].

#### 2.6.6. Melatonin and Lead-Induced Neurological Damage

Melatonin has been used in several situations to overcome lead toxicity. In rats, melatonin almost completely reduced the neurological damage caused by lead, thus exerting an important neuroprotective action by restoring the endogenous levels of GSH and SOD throughout the brain [29]. Furthermore, melatonin exerted a neuroprotective action, also acting positively on structural damage and on the reduction in neuronal density caused by lead. In addition to its important antioxidant action, further mechanisms involved in neuroprotection have been described, such as interaction with calmodulin, blocking increases in intracellular Ca^2+^, changes in gene expression, and the improved efficiency of mitochondrial oxidative phosphorylation. In cultured human neuroblastoma cell line SH-SY5Y, melatonin also restored lead-reduced GSH levels and protected against apoptosis by inhibiting caspase-3 activation [30].

### 2.7. Metal-Chelating Activity of Melatonin: Concluding Remarks

**Figure 4:**
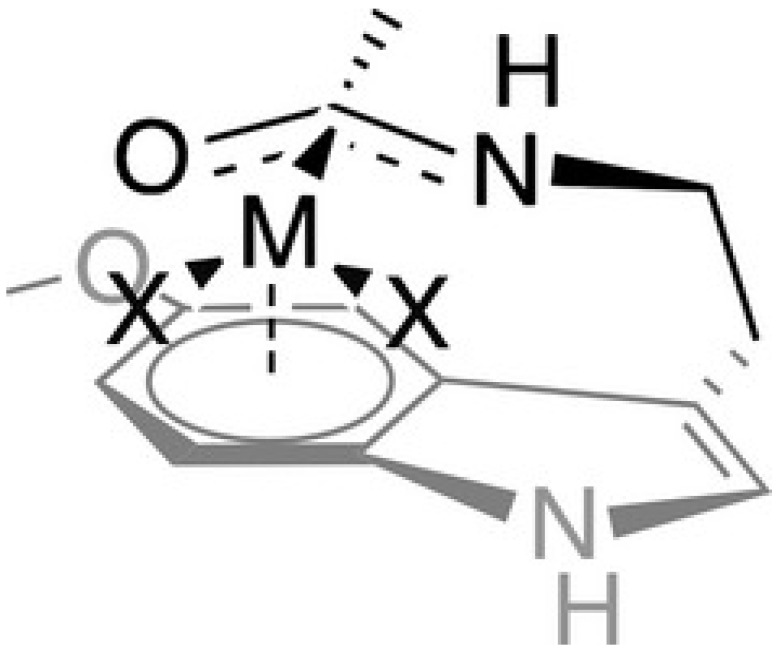
Hypothetical η6-coordination of metals to melatonin through the π-electron density of the benzene-fused ring in a tetrahedral-like fashion. From Romero et al. [12], with permission.

The military, due to their peculiar work activity, can be exposed to heavy metals. For this reason, logistical strategies should be implemented to reduce or eliminate possible sources of occupational exposure to heavy metals. However, especially in military operations abroad, this is not always controllable. In this context, the administration of melatonin can represent a very interesting primary prevention strategy against professional exposure to heavy metals, both for its beneficial biochemical actions, and also due to its very high pharmacological safety profile. Melatonin is a non-enzymatic antioxidant (i.e., the antioxidant H) able to deactivate the ability of heavy metals that trigger oxidative processes [31]. The melatonin/metal complex follows the organometallic rules for ligand–metal coordination, that is, the rule of the 18 electrons (16 electrons for ‘platinum metals’, such as Ni^2+^ complexes) and the generation of tetrahedral, octahedral, or square planar structures. A hypothetical schematic of the possible melatonin/metal bond is shown in Figure 4. The accumulation of metals in cells triggers epigenetic changes, abnormal cell signaling, uncontrolled cell growth, the initiation of cell damage, and the stimulation of inflammatory processes. Melatonin is a valuable protector against metal-induced damage thanks to its multiple properties, including its high lipophilicity that makes it easily transportable through cell membranes and its very low toxicity.

### 2.8. Melatonin for Protection from Chemical Weapons

Chemical warfare agents (CWAs) are substances that incapacitate a person and can kill them, mainly causing neurological damage, but also to the destruction of other organs. Currently available decontamination methods make it essentially impossible to quickly decontaminate a site after CWA exposure. Additionally, there are no completely effective antidotes or treatments against these agents after individuals are exposed to CWAs. Considering the complexity of the physiological processes that occur after exposure to CWAs, melatonin may be a useful antidote and may provide a reasonable strategy to counteract CWA-induced injuries [32].

### 2.9. Melatonin against Mustard Gas Toxicity

Oxidative stress and intracellular molecular damage are the main pathogenetic mechanisms behind the toxicity of blister agents such as mustard gas, chlorine, and phosgene. An interesting in vivo study evaluated the role of melatonin as an antidote in acute mustard gas intoxication. The most reliable parameter to test the serious nature of the exposure was the level of total plasma protein carbonyls. The carbonyl levels were significantly increased due to mustard gas contamination, and carbonylation was slowed due to melatonin intake. Carbonyl groups are a generic oxide production of biomolecules, and their formation on proteins is considered a severe, oxidative, and generalized stress marker. Melatonin has proven to be a prospective compound for reducing mustard gas toxicity damage in rats [33].

#### 2.9.1. Mustard Gas as an Invalidating Agent: The Role of Melatonin in Acute Toxicity

Sulfur mustard (SM), also known as mustard gas, is a widely used chemical agent. SM is used as a disabling and incapacitating agent rather than to cause death. Melatonin has been shown to be useful in acute MS toxicity through various mechanisms of action, including the ability to repair DNA damage and restoring the correct production of cellular energy at the mitochondrial level.

#### 2.9.2. Mustard Gas as an Invalidating Agent: The Role of Melatonin in Chronic Toxicity

The pathogenetic mechanisms behind the delayed toxicity of mustard gas (i.e., the harmful effects that occur over time after acute exposure) are not particularly clear. However, the epigenetic perturbations caused by MS appear plausible [34]. The term epigenetic describes the possibility of changes in gene expression caused by environmental factors in the absence of changes in the genome sequence. Consequently, the biochemical–molecular mechanisms that encode information beyond the basic DNA sequence and that can be transmitted through mitosis and meiosis form the basis for epigenetic gene regulation. Consequently, the biochemical–molecular mechanisms that encode information beyond the basic DNA sequence and that can be transmitted through mitosis and meiosis form the basis for epigenetic gene regulation.

### 2.10. Melatonin for Protection from Chemical Weapons: Concluding Remarks

**Figure 5:**
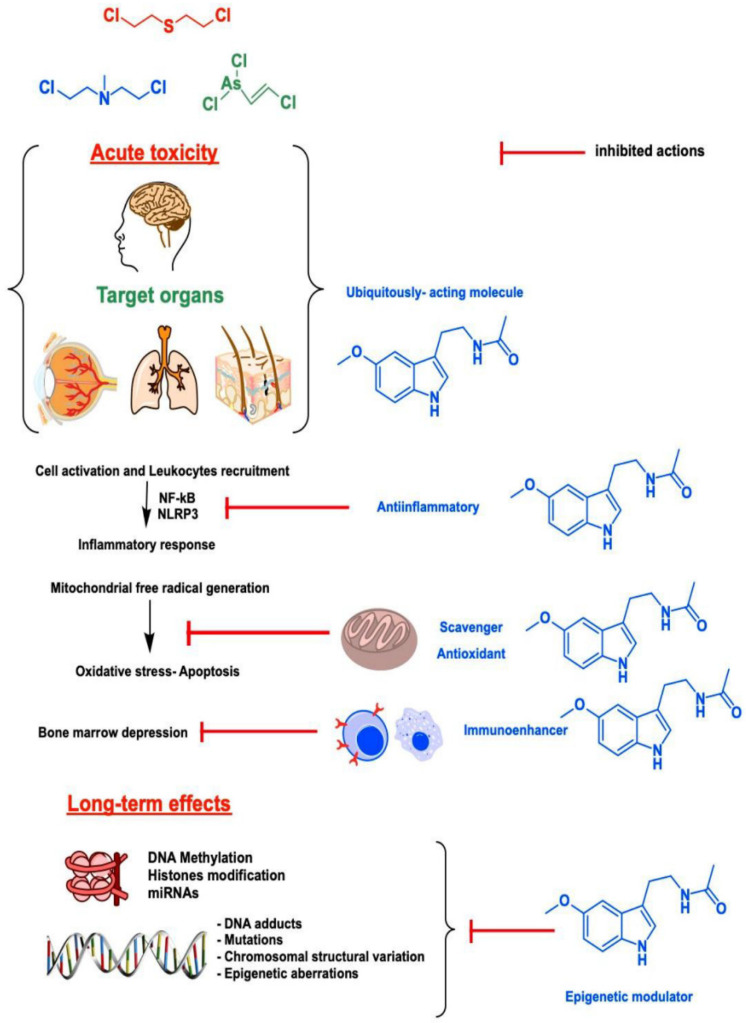
Summary of the cellular and molecular mechanisms displayed by melatonin against vesicant chemical warfare agents (CWAs). From Romero et al. [38], with permission.

Current knowledge on epigenetic regulation mainly focuses on two molecular mechanisms: histone modification and DNA methylation. It should be remembered that the beneficial action of melatonin in patients with advanced cancer also seems to derive from the combined effects of these two epigenetic mechanisms. In an in vitro study, the data showed that, in high concentrations, melatonin modulates P53 and Bax/Bcl-2 expression [35]. In addition, it is presumed that melatonin inhibits DNA methyltransferases (DNMTs); this is a family of enzymes that methylate DNA at the carbon-5 position of a cytosine residue [36]. There is evidence of the direct epigenetic actions of melatonin on various cellular targets, including histone acetylation enzymes [37]. Melatonin significantly increased mRNA expression for various HDAC isoforms and histone H3 acetylation in neural stem cell lines. Melatonin, as a whole, has non-genomic, genomic, and epigenetic actions. These important biochemical actions of melatonin can be very useful for both acute and delayed mustard toxicity. Romero et al. [38] summarized the cellular and molecular mechanisms displayed by melatonin against CWAs (Figure 5).

#### Chemical Agents as War Weapons and the Potential Use of Melatonin

Chemical agents have been used in war, with particular use during World War I. However, there are numerous examples of the use of this type of weapon in modern times (Japan 20 March 1995, Iraq–Iran war, Afghanistan war, etc.). For this reason, in the military but also in the civil field, it is necessary to study and develop new and increasingly efficient protection systems. Fortunately, melatonin already exists, and we can use it in integrated protection/prevention and treatment approaches in cases of acute and chronic chemical weapon poisoning.

### 2.11. Melatonin as a Radioprotective Agent

Ionizing radiation causes harmful effects to cells through both direct and indirect mechanisms. The cellular molecules sensitive to this form of radiation are damaged by direct action. The indirect effects—which represent about 70% of the overall damage—consist of a pathological interaction with the water molecules that determine the formation of important quantities of free radicals, such as •OH, •H, and eaq−. This, in turn, causes alterations in the subcellular structure. Due to its remarkable antioxidant capacity, melatonin represents an important molecule with radioprotective function. Many studies, both in vitro and in vivo, have confirmed that melatonin protects mammalian cells from the toxic effects of ionizing radiation. Furthermore, numerous clinical studies in the oncology field have documented that the administration of melatonin, also in combination with radiotherapeutic agents, determines a favorable efficacy/toxicity ratio in the treatment of human cancers [39]. It is believed that most of the tissue damage caused by ionizing radiation (about 60–70%) is attributable to •OH, which melatonin quickly neutralizes [40].

### 2.12. Melatonin Reduces Genetic Damage from Exposure to Ionizing Radiation

Among the DNA bases, guanine is the most susceptible target to oxidative damage mediated by free radicals, including •OH. In addition to DNA, free radicals also interact with lipids, causing the formation of hydroperoxides [41]. In recent years, the •OH-scavenging ability of melatonin has been tested to evaluate the radioprotective capacity of this molecule. In vivo studies have demonstrated the efficacy of melatonin as a radioactive protector in a dose-dependent manner. For example, Vijayalaxmi et al. observed that the exposure of CD2-F1 mice to 815 cGy of ionizing radiation (LD50/30 dose that kills 50% of mice in 30 days) resulted in a survival rate of 45–50% after 30 days; pretreatment with melatonin at a dose of 125 mg/kg bodyweight increased survival to 60%, while melatonin at a dose of 250 mg/kg bodyweight further increased survival to 85% [42].

### 2.13. The Role of Melatonin before Exposure to Radiation

This group also found that mice pretreated with 5 or 10 mg/kg of melatonin 1 h prior to radiation exposure showed significantly reduced genetic damage in bone marrow cells, with the 10 mg dose being more effective than the 5 mg dose [43]. The scavenging efficiency of melatonin, together with its indirect antioxidant properties, is documented in numerous independent research articles (over 900 publications in the literature).

### 2.14. Melatonin as a Radioprotective Agent: Concluding Remarks

As soon as exposure occurs, people—even if they live far from the primary event—could protect themselves through the oral administration of melatonin. This can be repeated several times depending on the specific case and under medical indication. Finally, the toxic side effects of melatonin are none or minimal at most. It is important to evaluate the full potential of melatonin as a radioprotective agent in day-to-day life, as well as in extraordinary conditions, including military settings in the event nuclear weapon use.

## 3. Melatonin for Psycho-Physical Performance of Flight Force

### 3.1. Melatonin and Cardiovascular Oscillations

Maintaining physiological cardiovascular oscillations is of great importance for maintaining optimal health. Simultaneous recordings of arterial pressure (AP) and sympathetic nervous system (SNA) activity in both animals and humans through spectral techniques revealed the presence of spontaneous oscillations of the aforementioned signals at slower frequencies than respiratory movements. Of particular interest are the so-called Mayer waves (corresponding to the “10-s rhythm” or 0.1 Hz in humans). The Mayer wave can be defined as the physiological oscillation of arterial pressure (AP) in synchrony with the sympathetic nervous system (SNA) [44]. AP oscillations that satisfy this requirement have a characteristic frequency of ~0.1 Hz in humans [45]. A common feature of Mayer waves is that their frequency is fairly stable within a given species. In particular, it has been shown in humans that this frequency does not depend on sex, age, or posture [46]. These oscillations in the 0.1 Hz frequency range are mainly determined by the baroreceptor and chemoreceptor reflex control system [47]. A method for opening the baroreflex loop is to interrupt the sympathetic transmission at the vascular neuroeffector junction. The reflected origin of these waves has been demonstrated through the use of phentolamine as an antagonist of alpha-adrenergic receptors, which strongly depressed both the Mayer wave and the oscillations of the autonomic nervous system [48].

### 3.2. Melatonin’s Epigenetic Actions

Hemodynamic oscillations related to Mayer waves have been demonstrated in human cerebral circulation [49]. The pineal gland, by means of its secretory product melatonin, has been implicated in the modulation of the cardiovascular system. Melatonin improves the baroreflex response in correlation to its antioxidant effects [50]. Melatonin influences vascular reactivity [51]. MT1 receptor activation causes vasoconstriction; in contrast, MT2 receptor activation causes vasodilation [52]. The most interesting mechanism by which melatonin can improve physiological cardiovascular oscillations is probably its epigenetic action on the adrenal glands and the heart. Melatonin modifies the genetic expression of NR3C1, the glucocorticoid receptor, in the adrenal gland and the heart [53]. In particular, melatonin reduces the NR3C1 gene in the heart and increases it in the kidneys and lungs. This has several clinical implications, such as increased resistance to hypoxia and improved neuro–cardio–respiratory resonance.

### 3.3. NR3C1 Gene and Stress Management

In mice, blocking the glucocorticoid receptor in cardiomyocytes and vascular smooth muscle cells resulted in major changes in the structural, functional and biochemical maturation of the fetal heart, thus indicating that the glucocorticoid signaling pathways activated by this receptor are vital for the normal structural and functional development of the heart [54]. In a noteworthy in vivo study on full-term infants, the methylation of the NR3C1 gene was found to be associated with an increase in cardiac variability expressed as RSA (respiratory sinus arrhythmia), and therefore, they had a greater ability to manage stressful events [55].

### 3.4. The Heart as a Door to Evaluate the Autonomic Nervous System

Heart rate variability (HRV) is a widely used parameter to assess autonomic nervous system (ANS) activity and balance between the sympathetic and parasympathetic branches. One measure of HRV is respiratory sinus arrhythmia (RSA), which identifies heart rate variability that coincides with breathing and reflects parasympathetic control [56]. Prolonged exposure to stress is associated with an autonomic imbalance, that is, a hyperactive sympathetic system and a hypoactive parasympathetic system [57]. NR3C1 is probably the most studied gene in human behavioral epigenetic research. There are a number of reports describing the differences in DNA methylation of the NR3C1 gene to cortisol reactivity and the hypothalamic–pituitary–adrenal (HPA) axis response [58]. Epigenetic modifications of NR3C1 can affect the autonomic nervous system; particularly, an increased methylation of the NR3C1 exon 1F at CpG sites 12 and 13 may be associated with an activation of the parasympathetic pathways with a consequent increase in RSA. It would be interesting to evaluate the possible effects of the exogenous administration of melatonin on cardiac variability expressed as RSA. Historically, melatonin has been repeatedly shown to impact heart and cardiovascular function. A more efficient autonomic modulation may have a positive impact on the psycho-physical performance of military personnel in terms of the prevention of chronic degenerative pathologies and also in terms of a better response to complex stimuli.

### 3.5. Melatonin and Heart Rate Varibility—HRV

**Figure 6:**
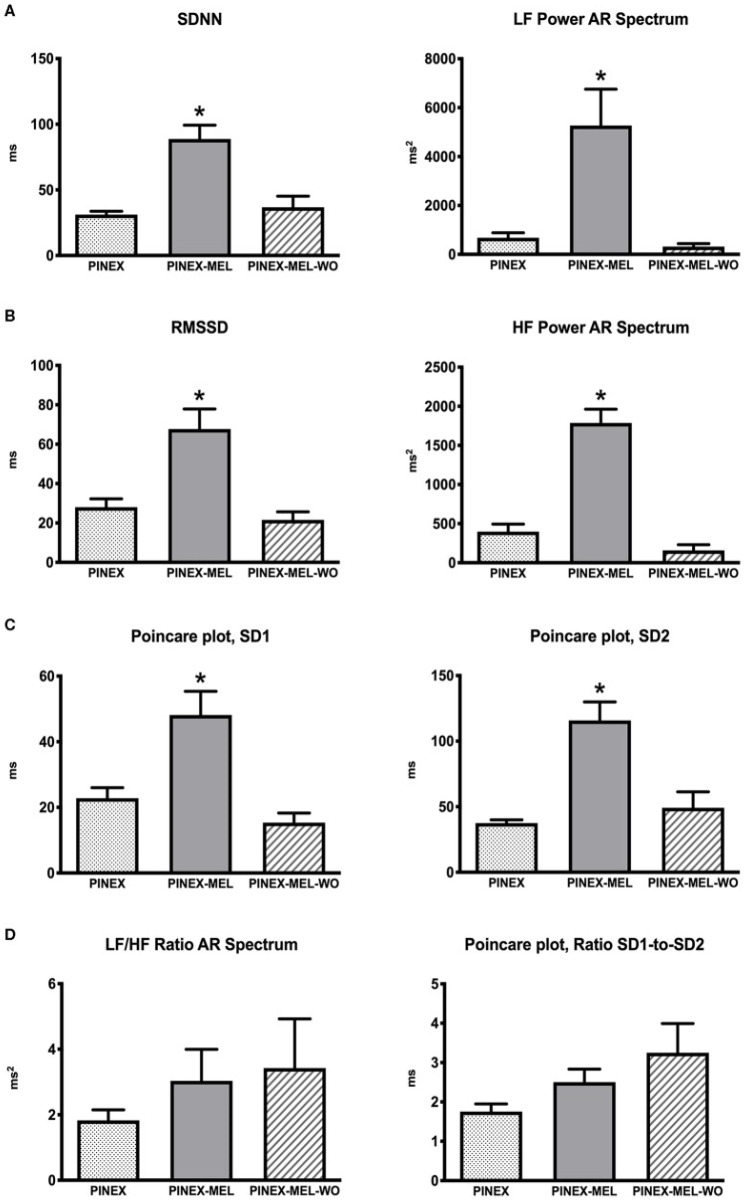
(**A**) Sympathetic modulation of heart rate variability (HRV) +/− the standard deviation of the normal R-R wave interval (SDNN). (**B**) Parasympathetic modulation measures: root mean square of the successive R-R interval differences (RMSSD). (**C**) Parasympathetic modulation: Poincaré plot analysis measures of SD1 and SD2; * *p* < 0.05 significantly different from the other groups. (**D**) Autonomic balance estimated through the ratio of SD1 to SD2 of Poincaré plot and LF/HF ratio of AR spectrum. From Campos et al. [64].

Heart rate variability (HRV) can be defined as the physiological variation in the time interval between heartbeats. Both heart rate and cardiac output are influenced by the efferent vagus nerve [59]. HRV can be assessed by time domain, frequency domain, and nonlinear variables [60]. The standard deviation of the RR intervals (SDNN) is used as an overall estimate of autonomic function. The square root of the mean squared difference between the adjacent RR intervals (RMSSD) is predominantly influenced by vagal tone [61]. SDNN is an index of general health while RMSSD is an index linked to the cholinergic anti-inflammatory pathway (CAP) [62]. These parameters show robust circadian rhythmicity, and melatonin has been studied as a possible intervention to modulate the autonomic nervous system [63]. The published findings show that treatment with melatonin for 3 months (3 mg/day) induces an improvement in cardiac autonomic modulation in melatonin-non-proficient patients [64] (Figure 6).

### 3.6. Melatonin for Autonomic Modulation: A Powerful Strategy for Militaries

A recent study examined the effects of melatonin (2 mg/day) administration on heart rate variability (HRV) in 26 healthy men. The findings indicate that melatonin administration increases cardiac vagal tone in the supine position in awake men. Melatonin administration also seems to exert suppressive effects on sympathetic tone [65]. This is extremely interesting data for the military, as low dosages of melatonin may be sufficient to supplement autonomic modulation and cardiovascular function. This is particularly significant as this molecule is extremely safe, even at very high doses. Taking melatonin in the evening facilitates an increase in vagal tone and, therefore, helps to create the physiological circadian response of the stress system (activating the parasympathetic system in the bathyphase to deactivate it adequately in the acrophase). Moreover, it facilitates an adequate psychophysical energy level to carry out the complex tasks that the military must deal with on a daily basis.

### 3.7. Melatonin to Improve Physical Endurance

Physical exercise, stimulating the sympathetic nervous system, can affect the secretion of melatonin. Melatonin is used as a natural supplement among athletes to regulate sleep cycles and protect muscles from oxidative damage due to the type and intensity of the activity performed. Exercise ≥ 50% VOmax2 induces an increase in ROS/RNS concentrations above physiological levels. The production of these harmful agents depends on various factors (e.g., determinants of exercise, postural position during exercise, training state, age, sex, and diet) [66]. Prolonged intense training also reduces an athlete’s endogenous melatonin level [67]. Based on published data, the optimal way for elite athletes to take melatonin (presumably equally applicable for military personnel) is at a dose of 10 mg in the evening to exploit its antioxidant properties, improve sleep quality, and enhance physical performance.

### 3.8. Melatonin to Improve the Physical Performance of Military Personnel

Large doses of melatonin taken alongside physical activity may hinder performance, primarily due to its effect on sleepiness and sympathetic depression [68]. However, in cases of sleep disturbance or jet lag syndrome, the combination of exogenous melatonin and outdoor physical exercise (to increase exposure to natural light) is a means to improve performance in sports. Thus, the same can be assumed for military combat personnel.

## 4. Melatonin in Sepsis

The pineal gland is not the only source of melatonin; it is also produced in the mitochondria of many other cells, including in lymphocytes. Melatonin has a fundamental role in many biochemical functions, and its action in the course of viral and bacterial infections has also been evaluated. The rationale for the use of melatonin in this context not only derives from its antioxidant, immunomodulating, anti-inflammatory, and coagulation regulation abilities, but also on its direct inhibitory actions on pathogenic microorganisms, bacteria, and viruses. As already noted, melatonin is a very well-documented antioxidant, but it also exhibits pro-oxidant actions depending on the pathophysiological situation; this is particularly useful for its action against microorganisms. For example, its activity against equine encephalitis virus and the bacterium responsible for tuberculosis has been demonstrated in animal and in vitro models [69,70,71]. In addition, an action against chlamydial microorganisms has also been proven; these are responsible for respiratory problems and infertility in humans. The action of melatonin on these microorganisms is thought to be a consequence of an increase in interferon gamma induced by melatonin that is essential in the course of viral infections and beyond [72]. Finally, action on *Staphylococcus aureus* and *Pseudomonas aeruginosa* bacteria was also noted with a description of a variety of mechanisms of action by which melatonin reduces metabolic substrates important for bacterial growth. This action has been shown to be especially effective against gram (−) bacteria compared with gram (+) strains [73].

### 4.1. The Systemic Anti-Infectious Action of Melatonin

In summary, melatonin has the unique ability to resist systemic viral and bacterial infections, even in the case of sepsis, which can be a very serious condition. Some of the major mechanisms that explain the antiviral and antibacterial actions of melatonin are listed below [74]:

- Antioxidant actions in numerous cells with pro-oxidant actions on specific pathological targets;
- Anti-inflammatory functions with reductions in pro-inflammatory cytokines, including IL-6, TNF-alpha, and an increase in anti-inflammatory proteins, including IL-10;
- Inhibitory actions on bacterial growth through the removal of biochemical elements from microorganisms useful for multiplication;
- Maintenance of an adequate mitochondrial enzymatic profile, inter alia, at the level of respiratory cells.

Several clinical studies have been carried out to evaluate the role of melatonin in the course of septic shock and multi-organ failure, which often leads to the death of the diseased patients. Of 302 patients admitted to intensive care with sepsis who were in danger of developing multi-organ failure, a clear correlation between melatonin deficiency and disease severity emerged, so much so that the authors concluded that melatonin supports the body’s defenses during septic shock [75]. Studies have been carried out on newborns suffering from sepsis and chronic lung diseases, with melatonin administered at high doses (from 20 to 80 mg per day) to evaluate their blood chemistry, inflammatory parameters, and clinical evolution. In both studies, none of the children treated with melatonin died, while in the placebo groups, there were unfortunately five deaths in total. The oxidative and inflammatory parameters (IL-6, TNF-alpha, IL-8) were significantly reduced in the individuals treated with melatonin, and these aspects were indicated as responsible for the positive clinical evolution of the treated children [76,77,78].

### 4.2. Melatonin for the Prevention of Septic Complications and Multi-Organ Insufficiency

In an RCT clinical study carried out on 24 patients with tracheostomy at a dosage of 10 mg per day for 4 consecutive days, in addition to the expected improvement in sleep quality, a preventive action against septic complications and multi-organ failure as well as a reduction in ischemic lesions due to tissue reperfusion emerged [79].

### 4.3. Melatonin as a Support on the Battlefield

In field conditions in foreign environments, military personnel are routinely exposed to a variety of potential pathological viral and bacterial organisms; soldiers who develop symptoms of such infections would likely be incapable of carrying out their military responsibilities. Experience in the field shows that sepsis is not an overly frequent event (occurring in about 1% of wounded soldiers), especially if the evacuation from the battlefield takes place without delay; however, sepsis has been shown to reduce survival rates, for which melatonin certainly represents a valid support on the battlefield.

## 5. Extending the ‘Golden Hour’ to Improve Medical Outcomes

The phrase ‘golden hour’ refers to the duration of time elapsed between a severe injury being sustained and in-hospital care being initiated. In this phrase, the term ‘hour’ is arbitrary and can be of any duration, but usually the shorter this interval is, the better the chances are of reduced morbidity and patient mortality [80,81]. In reference to remote battlefield situations, this so-called ‘golden hour’ is often significantly prolonged for a variety of reasons. While there have been a number of significant advancements in handling these combat-related emergencies, which have improved the survival of severely injured individuals, there is always a need to further reduce the elapsed time from trauma to optimal treatment or to provide better on-site immediate care. Although the duration of the ‘golden hour’ does not always correlate with morbidity or mortality [82], the early transport of injured individuals to fully equipped medical facilities via helicopter emergency medical service (EMS), or another means, often improves survival [83]. At a minimum, for the severely injured combatant, it is psychologically reassuring to undergo treatment in an intensive care hospital setting as opposed to on the battlefield.

### 5.1. Critically Traumatized Patients: ‘Scoop and Run’ or ‘Stay and Treat’?

Procedures that enhance the survival of those suffering severe blood loss include massive blood transfusions and resuscitative endovascular balloon occlusion of the aorta. There is a debate as to whether EMS care providers should ‘scoop and run’ or ‘stay and treat’ critically traumatized patients [84]. Under battlefield conditions, this becomes an even more significant factor given that the situation may preclude the early transfer of the patient by helicopter or land vehicle. Moreover, the ‘scoop’ or ‘stay’ scenarios may be influenced by the on-site treatment resources available to the EMS technicians, as well as to the nature and severity of the tissue damage [85]. Based on the subjective impression of the on-site medics, whether a patient is considered unstable/critical is also a consideration since these subjects usually demand a shorter pre-hospital interval compared with subjects who are considered stable [86].

### 5.2. Massive Bleeding: The Principal Target of Severely Traumatized Patients

Excessive blood loss, which leads to hemodynamic instability, is often a factor in determining the survival of severely traumatized patients [87]; on the battlefield, this may be a result of traumatic amputations, multiple wounds, etc. Excessive systolic hypotension (i.e., between 70 and 90 mmm Hg) is classified as ‘permissive hypotension’ and is sometimes beneficial since it reduces the likelihood of the dislodgement of blood clots when the subject is being treated [88]. In the short term, permissive hypotension may improve survival, but when prolonged, it leads to tissue hypoxia, high free-radical generation, and severe tissue damage with the potential development of multiple organ failure and death [89,90]. As indicated, excessive hemorrhage contributes to an increased risk of death.

### 5.3. Melatonin as Support for Massive Bleeding

In the context of the military environment, hemorrhagic shock is a common feature associated with traumatic injury and is a frequent cause of battlefield death, especially when the victim does not have timely access to comprehensive medical care [91]. Considering the serious nature of this problem, attempts have been made to develop supplemental technologies that could be useful in the battlefield situation to aid in the resuscitation and maintenance of individuals suffering from profound blood loss. Perez de Lara Rodriguez and colleagues [92] used a rat model in which 60% of the blood volume was quickly removed, simulating the serious hemorrhage that accompanies a devastating injury; the specific aim of this study was to test molecules that may prolong the ‘golden hour’. This group had earlier developed this model as a system in which to test compounds that may improve the survival of animals suffering from severe blood loss [93]. When these hypotensive animals (mean arterial blood pressure of 25 mm Hg) were treated with one small bolus injection (1 mL/kg) of melatonin combined with beta-hydroxybutyrate (BHB), the mean survival time was significantly prolonged. An injection of BHB only had a minimal effect on survivability.

### 5.4. Melatonin to Produce Extra ATP during Massive Bleeding

The authors identified several actions of these molecules that may explain their ability to prevent the early death of the animals experiencing a hemorrhagic shock. Similarly to glucose, BHB serves as a fuel source for cells, but it does not generate lactate; it enhances mitochondrial acetyl-co-enzyme A (acetyl co-A) synthesis, which, via the tricarboxylic acid cycle (TCA), amplifies energy (ATP) production. Not mentioned by the authors is the fact that melatonin also improves ATP production directly by preserving the efficiency of the enzymes of the electron transport chain [94]. Additionally, of great potential importance is that acetyl co-A, besides feeding into the TCA cycle, is a required substrate for mitochondrial melatonin biosynthesis [95]. This likely has great importance since this alternate source of melatonin further promotes mitochondrial energy generation, while also functioning as an antioxidant to reduce oxidative damage to cardiolipin and to the enzyme proteins of the ETC, thereby further supporting ATP synthesis [96,97]. The extra ATP is surely related to the improved survival of the animals suffering from hemorrhagic shock, since cells cannot survive without an adequate energy supply; thus, these drugs have potential utility in extending the ‘golden hour’.

### 5.5. Melatonin Improves the Survival of Animals during Massive Bleeding

The multiple ROS-scavenging actions of melatonin and of its metabolites are also of relevance to survival and long-term outcome, as discussed elsewhere in this review (see above). Studies related to the ability of melatonin and BHB to enhance the survival of animals suffering from hemorrhagic shock were extended to pigs by Mulier et al. [98] and by Wolf and colleagues [99]. The results again showed that melatonin administration improved the survival of pigs experiencing massive blood loss. Pigs are often used in such experimental studies because they are considered the best available model for humans.

### 5.6. Melatonin to Extend the ‘Golden Hour’: Concluding Remarks

The results of these investigations established that a bolus injection of 43 mM melatonin combined with 4 M BHB provides the best protection against hemorrhage shock in terms of survival in this preclinical model. Both of these drugs have anti-inflammatory and antiapoptotic actions, which bodes well for the quality of life of the survivors of massive blood loss [100,101]. Collectively, the data reviewed in this section strongly support human trials using melatonin alone or in combination with BHB to determine its/their utility for improving the survival of individuals who have suffered significant blood loss. Animal studies have shown that the use of these drugs prolongs survival after extensive hemorrhage and thereby could extend the duration of the ‘golden hour’ prior to a wounded soldier’s arrival at a more comprehensive medical facility. Positive results in these trials would have clear implications for the battlefield situation since melatonin is a highly stable molecule and could easily be immediately administered under field conditions without worrying about significant toxicity or untoward side effects. No lethal dose of melatonin has been identified.

## 6. Reducing Oxidative Stress and Preserving Organ Function

Extreme hypotension resulting from a marked reduction in blood volume has cellular consequences, which contribute to tissue damage and cellular malfunction in every organ. Importantly, reduced blood flow leads to relative hypoxia in the downstream organs. Cellular hypoxia activates signaling pathways that cause the excessive generation of partially reduced oxygen derivatives (i.e., ROS or free radicals). These toxic agents oxidize critical molecules (lipids, proteins, RNA, DNA), which compromises the optimal function of subcellular organelles, thereby initiating cell death. This damage can occur in any organ, but it is especially common to cells that rely especially heavily on oxygen availability (for example, neurons and cardiomyocytes). Since these cells are not renewable, unlike many others, their loss is highly significant, and, when hypoxia depresses the function of several vital organs, it contributes to multiple organ dysfunction syndrome (MODS) and an elevated mortality [102]. Reducing the likelihood of MODS is essential to improving the survival of combat-injured soldiers. In experimental and clinical situations, melatonin reduces multiple organ failure and the death of animals and humans [103,104]. The protective actions of melatonin against MODS are a result of its multiple anti-inflammatory and antioxidant properties [105].

### 6.1. Melatonin as an Inhibitor of High Mobility Group Box 1 (HMGB1)

An essential component of intensive inflammatory processes is the activation and release of the multifunctional high mobility group box 1 (HMGB1) protein [106]. Intensive research has been directed at identifying drugs to inhibit HMGB1 [107] for the purpose of reducing organ damage and minimizing dysfunction. In a variety of preclinical models, melatonin is confirmed as a potent inhibitor of HMGB1 activation resulting from inflammation and oxidative stress [108]. This suggests that tissue damage in severely injured combat personnel where uncontrolled inflammation and excessive oxidative stress are usual would be reduced by melatonin administration, which could contribute to a healthier outcome. In addition to blood loss and the associated hypoxia, which initiates oxidative stress, many other processes where hypoxia is not necessarily a factor also cause damage to subcellular organelles, resulting in the production of elevated ROS/RNS without adequate removal. The loss of redox homeostasis is a major cause of endoplasmic reticulum and mitochondrial physiology perturbations, which contribute to cellular dysfunction and eventually disease initiation and progression [109,110].

### 6.2. Melatonin Reduces the Molecular Damage Induced by Oxidative and Nitrosative Stress

Oxidative/nitrosative damage to any organ can result in acute disabilities with potential long-term consequences. In reference to military personnel in battlefield situations, organs that are especially vulnerable include skin, eyes, and lungs. Damage to these organs results from toxin exposure and environmental pollutants, including mustard gas, particulate matter in smoke and soot, airborne heavy metals, exposure to viruses and bacteria [111,112,113,114], and a result of physical harm, for example, contusion [115,116]. The oxidatively-mediated molecular damage resulting from these destructive agents is known to be mitigated by melatonin [117,118,119].

### 6.3. Melatonin as a Molecule That Promotes and Synergizes with Other Antioxidants

Melatonin’s efficacy in situations where oxidative stress is a major parameter of tissue damage and disease stems from its broad-spectrum antioxidant actions. Melatonin and its metabolites are not only direct scavengers of ROS/RNS; they also promote antioxidative enzymes and synergize with other better-known classic antioxidants to neutralize partially reduced species or to metabolize them to innocuous by-products [120,121]. Many of these actions are most obviously manifested at the level of the mitochondria, which are major sites of toxic radical generation [122,123] and are important organelles for maintaining optimal cellular physiology.

### 6.4. Melatonin as a Protective Agent in Neurological Damage

A significant form of injury experienced during military deployment is concussion resulting from blast trauma, which varies from mild to extremely severe. These injuries are particularly devastating when they involve the brain, which they often do [124,125]. When the CNS is involved, such damage is referred to as traumatic brain injury (TBI); the neural alterations are a consequence of the direct energy transfer of a shockwave [126]. Although there is still much to be learned about the molecular events that initiate TBI, signature processes resulting from such damage are perturbations of mitochondrial physiology, including impaired bioenergetics, faulty oxidative phosphorylation, and elevated oxidative stress [127,128]. Because of the post-concussive symptoms of these injuries, they significantly compromise the subsequent quality of life of these individuals. Some long-term disabilities include cognitive and memory deficits and often a significant increase in the likelihood of developing dementia [129]. There are no Food and Drug Administration (FDA)-sanctioned therapies for the treatment of TBI. Melatonin has been tested as a protective agent against neurological damage in many preclinical models of traumatic injury to the CNS [130]. Despite the positive findings regarding the beneficial effects of melatonin against TBI in experimental animals, its use for this purpose in humans remains woefully under-investigated; for example, in one study that was published, the dose of melatonin was minimal (3 or 10 mg), and it was only given once per day at bedtime [131].

### 6.5. Melatonin in the Treatment of Traumatic Brain Injury: In Vivo Studies

The animal studies summarized below indicate that melatonin is effective in reducing neuronal damage when administered continually for the first 4 h after TBI, and in larger doses than used in the published human trials. Moreover, in the case of human studies, actual neuronal survival has not been evaluated. It is essential that, to exhibit efficacy, the dose of melatonin must be compatible with the severity of the TBI damage.

### 6.6. Mitochondria Are the Principal Target of the Action of Melatonin in Traumatic Brain Injury (TBI)

The experimental reports dealing with the use of melatonin to attenuate molecular damage to the CNS or to reverse the neurobehavioral consequences of TBI are not extensive in number and generally lack information regarding the survival of melatonin-treated TBI animals. Given the prominent involvement of the mitochondria in TBI-related pathophysiology, molecules that correct the physiology of these dysfunctional organelles may be useful as treatments for TBI. This was the rationale of Salman and colleagues [132] who used melatonin to arrest neurological damage in a rat model of TBI. To simulate a real-life situation, melatonin was given at intervals of 30 min up to 4 h post-TBI; the administered dose was 10 mg/kg, and it overcame oxidative stress and mitochondrial dysfunction. The mitochondrial parameters measured showed improvement in the brain of TBI-treated rats as a result of melatonin treatment. The melatonin-mediated changes included improved energy production, a reduction in neuronal apoptosis, and the promotion of mitochondrial fusion; these improvements were accompanied by the increased expression of PGC-1α and Mfn2, both of which support mitochondrial biogenesis and would be expected to preserve neuronal and glial survival. As the central role of neuronal apoptosis is responsible for the secondary and residual brain dysfunction that follows a concussive injury to the head, Wu and colleagues [133] specifically examined the ability of melatonin to prevent neuronal loss following TBI. After subjecting rats to a controlled impact model of TBI, parameters of neuronal death were examined in the umbral and penumbral areas of the lesioned brain. When the animals were repeatedly given melatonin (10 mg/kg) up to 4 h after lesion induction, this reduced brain edema and attenuated neuronal apoptosis. Moreover, it limited the neurobehavioral deficits, as shown by an improved performance in motor, sensory, and balance tests. Likewise, in mice, treatment with melatonin (10 mg/kg bodyweight daily) after direct controlled cortical impact injury suppressed lipid peroxidation, reduced neuronal loss, and relieved endoplasmic reticulum stress in the damaged area of the brain [134].

Finally, restoring damage to the CNS after TBI is improved by injecting neural stem cells into the lesioned area. Typically, stem cells do not survive well under these conditions, but their injection in a solution also containing melatonin significantly enhances their survival and thereby helps to regenerate normal neural morphophysiology [135].

### 6.7. Melatonin in Injuries of the Spinal Cord

As with the intracranial portions of the CNS, the spinal cord is also highly vulnerable to damage, especially to crush injury. If severe, such damage can permanently incapacitate an individual, and research into methods/drugs to improve recovery are under intensive investigation [136]. Samantaray and colleagues [137] used a standard weight drop technique to the exposed rat spinal cord during laminectomy to simulate a crush injury. High-dose melatonin (45 mg/kg) given 15 min post-damage ameliorated the severity of all parameters measured in the injured region of the spinal cord; these included inflammation, axonal damage, calpain expression, and neuronal death. Similar beneficial effects of melatonin in a severe spinal cord crush injury have recently been reported [138]. Both of these groups proposed that melatonin has promise as a drug to alleviate the neurobiological consequences of spinal cord injury.

### 6.8. Melatonin in Neural Injuries: Concluding Remarks

Melatonin’s protective actions in the CNS, in models other than TBI or spinal cord crush injury, are also numerous [139,140]. Exogenously administered melatonin easily crosses the blood–brain barrier and enters the neurons and glia [141] and is described as being specifically targeted to the mitochondria where it accelerates energy generation, neutralizes ROS/RNS to protect these organelles from nitro-oxidative stress, and influences mitochondrial biogenesis [142,143]. The collective data overwhelmingly indicates that melatonin is a capable and essential protector of the CNS against any condition that depresses mitochondrial physiology because it deprives these organelles of the energetic substrates and oxygen required for their optimal function. In view of this, trials that take into account its timely utilization in pharmacological doses would very likely prove melatonin’s usefulness as a protective agent against the destructive effects of neural injuries experienced by military personnel.

## 7. Improving Damaged Soft Tissue Recovery and Bone Healing

Soft tissue healing is a highly complex physiological process that involves a plethora of mediators and four well-defined and highly programmed phases; these include hemostasis, inflammation, proliferation, and remodeling, and for optimal healing, these phases must occur in sequence [144,145]. Depending on the tissue and type of lesion, successful wound healing can require 6 weeks or more. In addition to debridement and primary closure, the wounds must be protected from contamination by particulate matter, viruses, and bacteria. Many advances have been made in the last two decades that have hastened wound healing and improved outcomes [146,147].

### 7.1. Melatonin to Improve Injured or Fatigued Muscle

The use of melatonin to assist in wound healing has been proposed by a number of investigators. The ability of melatonin to repair rat skeletal muscle damaged using a crush injury model was investigated by Stratos and co-workers [148]. After crushing the soleus muscle, melatonin was given daily at a dose of 10 mg/kg for the duration of the study; this treatment improved fast twitch and tetanic muscle strength post-injury. Additionally, the inflammatory response of the muscles was reduced, and, importantly, the number of satellite cells capable of forming new muscle fibers increased. Moreover, the number of apoptotic cells was reduced. The data uncovered supports the use of melatonin to regenerate the morphological and functional capacity of injured muscle resulting from a crush injury. Additionally, it may be able to improve recovery from muscle fatigue after intensive exertion.

### 7.2. Melatonin in Wound Healing

As with most soft tissue injuries, skin flaps often undergo at least partial necrosis, especially around the edges because of the initially compromised blood supply, which eventually leads to ischemia/reperfusion injury. In a series of in vitro and in vivo studies, Chen and co-workers [149] tested the efficacy of melatonin in reducing necrosis and improving the survival of repaired random-pattern skin flaps in rats. In the context of the current review, only the in vivo results are considered. Treating the animals with 40 mg/kg melatonin for seven days markedly prevented many of the unavoidable changes normally associated with skin flap restoration. The protection from melatonin was a result of its potent antioxidant and anti-inflammatory activities (see references [96,97,122]). These studies concentrated on examining mitochondrial malfunction in the skin flap cells, with all aspects showing improvement as a result of melatonin treatment. The molecular mechanisms by which melatonin preserved flap morphophysiology involved the activation of mitophagy, a reduction in oxidative damage, and the mitigation of apoptosis. These findings also have applications in skin transplants where a transient interruption of the blood supply is even more prominent. Providing melatonin to the patient receiving the transplant, as well as incubating the tissue in a melatonin-containing solution prior to attachment, would likely enhance the survival of the transplanted skin [150,151].

### 7.3. Melatonin in Burn Wound Damage

There is preliminary evidence that melatonin has benefits that limit burn wound damage. Data shows that, when given parentally, melatonin reduces cellular damage in the zone of stasis that surrounds the area of irreversible coagulation necrosis that is a result of a burn [152,153]. In particular, the histopathological examination of the zone of stasis revealed less edema and congestion, a reduced inflammatory response, and less fibrotic tissue generation, thereby limiting scar tissue. With the aid of a nanogel impregnated with melatonin, Soriano et al. [154] showed that its application to a burn injury not only improved epidermal growth but exhibited antimicrobial activity as well. Protecting burn wounds from microbes is an important aspect of successful treatment. There is also serious systemic multi-organ damage after extensive cutaneous thermal injury, including muscle wasting, inflammation, insulin resistance, and hypermetabolism. These should be considered targets for melatonin treatment given they are a result of compromised mitochondrial function, inflammation, and coagulopathies that are preserved by melatonin in other traumatic conditions and potentially after experimental thermal injury (see reference [153]).

### 7.4. Melatonin in Bone Marrow Stem Cell (BMSC) Therapy

There is a plethora of studies proving the efficacy of melatonin in speeding the healing of wounds when it is applied via a gelatin sponge [155], lecithin–chitosan nanoparticles [156], nanofiber wound dressing [157], chitosan-based microspheres [158], collagen/chitosan scaffolds [159], etc. Additionally, bone marrow stem cell (BMSC) therapy is being used successfully in several areas of regenerative medicine. When BMSCs were treated with melatonin and applied to the skin punch wounds of rats, all parameters of wound healing were further improved compared with treatment with non-melatonin-treated BMSCs [160]. Improvements were obvious from the histological measures as well as the molecular biology parameters, which included increased hydroxyproline levels, elevated expression of vascular endothelial growth factor (VEGF), and improved antioxidant activities in the regenerating tissues, among others. Moreover, another benefit of the use of melatonin in wound healing is attributed to its antibacterial actions [161].

### 7.5. Melatonin in Bone Fracture Repair

Bone fractures are common in combat situations. Given the pronounced effect of melatonin in promoting osteoblastic activity and suppressing osteoclastogenesis, it is expected that it would also be favorable in bone fracture repair [162,163]. One of the earliest studies that described the molecular mechanism by which melatonin supports fracture healing was published by Dong and co-workers [164]. Using a femoral fracture model in rats, they showed that treatment with melatonin physically improved the healing of the fracture, which was accompanied by an increased expression of neuropeptide Y (NPY) and NPY receptor 1 (NRY1R); treatment with a specific inhibitor of NPY1R prevented the beneficial effects of melatonin in fracture healing. In an associated study using cultured mesenchymal stem cells (MSCs), the addition of melatonin to the growth medium upregulated NPY and its receptor expression in MSCs undergoing differentiation into osteoblasts; a host of molecular biological measurements verified this. In a later study, this same group reported that the upregulation of osteoblast differentiation also required the stimulation of zinc transporter (Zip1) and documented its involvement in the NPY/NPY1 signaling pathway as well as osteogenic differentiation and femoral fracture healing [165].

### 7.6. Melatonin for Improving Bone Healing: Concluding Remarks

There are many other reports confirming the ability of melatonin to enhance bone formation, reduce osteoporosis, and aid in successful bone grafting [163,164,165,166]. For an extensively updated explanation of the molecular processes by which melatonin assists in bone metabolism and fracture recovery, the interested reader is directed to the recent publication of Huang et al. [167].

## 8. Concluding Remarks and Perspectives

It is very important to investigate in detail the role of melatonin in the synchronization of the neuro–cardio–respiratory systems and in the possibility of restoring, through its epigenetic action, the main oscillatory waves of the cardiovascular system, such as the Mayer wave and RSA (respiratory sinus arrhythmia), which, in physiological conditions, result in the oscillation of the heartbeat in synchrony with the breath. There are some considerations that are more philosophical–spiritual than scientific. The molecular structure of melatonin has never changed over the course of more than 3 billion years. This may mean that melatonin was born ready to protect, guide, and lead the evolution of every living species (vegetal and animal), including humans. As is well known, melatonin informs, via its circadian rhythm, every cell in the body of the light–dark environment, the major universal cycle. It serves as a bridge between us and the universe. In consideration of the rhythmic function of every biological system, all of which are influenced by the circadian melatonin rhythm, every attempt should be made to stabilize the light: dark cycle to which military personnel are exposed to maintain stable rhythms, thereby improving general health and well-being.

### 8.1. Melatonin to Extend the ‘Golden Hour’ on the Battlefield

Especially under battlefield conditions, time to treatment after an injury is often a major issue since the injured party may not have immediate access to medical care. Any drug that would help to stabilize a wounded individual, especially if it can be immediately administered (e.g., per os) and has a very high safety profile over a large range of doses, as melatonin does, would be an important asset to reduce morbidity and mortality. Indeed, if the wounded soldier is conscious and melatonin is available, the drug could be self-administered orally. This would be particularly relevant to mass causality and triage situations where medical aid is often further delayed. Any procedure, treatment, or medication that may prolong the so-called ‘golden hour’ for critically injured individuals should be considered in appropriate trials. Melatonin is a highly stable molecule, is inexpensive, can be easily and quickly produced in pure form, and does not require refrigeration. Thus, it could be made available to all members of a fighting force.

### 8.2. Melatonin to Allow the Soldier to Return to the Field Faster

In addition to potentially serving as a molecule to be immediately administered, there is also compelling experimental evidence that melatonin would have significant utility during the treatment and recovery periods. Much of the molecular damage sustained as a result of concussive, crush, and burn injuries involves the generation of an excessive amount of highly toxic free radicals, which mutilate and functionally incapacitate cells, making them vulnerable to undergoing apoptosis. Melatonin is a powerful direct radical scavenger and an indirect antioxidant when it promotes the expression of genes regulating antioxidative enzymes. Its functions are especially manifested in the mitochondria, organelles that are essential for optimal cell survival; at this site, melatonin also enhances ATP production, which improves cell recovery. Finally, melatonin is a powerful antiapoptotic agent.

### 8.3. Melatonin to Shorten the Healing Time of Soldiers

During long-term care, melatonin’s utility stems from its ability to enhance molecular processes that allow damaged tissues the greatest probability of successful recovery in the shortest possible time. This would be relevant in allowing soldiers to return to the field and would reduce overcrowding in the medical care unit. In addition, melatonin’s antiviral and antibacterial functions are important to protect wounds from infections that delay recovery. We urge the implementation of well-designed, randomized, double-blinded clinical trials to identify the fields of military medicine where melatonin may shorten time to recovery and improve clinical outcomes for injured military personnel.

## Data Availability

Not applicable.
